# Exploring different research questions via complex multi-state models when using registry-based repeated prescriptions of antidepressants in women with breast cancer and a matched population comparison group

**DOI:** 10.1186/s12874-023-01905-9

**Published:** 2023-04-10

**Authors:** Nikolaos Skourlis, Michael J. Crowther, Therese M‑L. Andersson, Donghao Lu, Mats Lambe, Paul C. Lambert

**Affiliations:** 1 Department of Medical Epidemiology and Biostatistics, Karolinska Institutet, Stockholm, Sweden; 2 Red Door Analytics, Stockholm, Sweden; 3 Institute of Environmental Medicine, Karolinska Institutet, Stockholm, Sweden; 4 Regional Cancer Centre Central Sweden, Uppsala, Sweden; 5 Biostatistics Research Group, Department of Health Sciences, University of Leicester, Leicester, UK

**Keywords:** Multi-state models, Prescribed drug register, Time until medication, Clock approaches, Flexible parametric models

## Abstract

**Background:**

Multi-state models are used to study several clinically meaningful research questions. Depending on the research question of interest and the information contained in the data, different multi-state structures and modelling choices can be applied. We aim to explore different research questions using a series of multi-state models of increasing complexity when studying repeated prescriptions data, while also evaluating different modelling choices.

**Methods:**

We develop a series of research questions regarding the probability of being under antidepressant medication across time using multi-state models, among Swedish women diagnosed with breast cancer (*n* = 18,313) and an age-matched population comparison group of cancer-free women (*n* = 92,454) using a register-based database (Breast Cancer Data Base Sweden 2.0). Research questions were formulated ranging from simple to more composite ones. Depending on the research question, multi-state models were built with structures ranging from simpler ones, like single-event survival analysis and competing risks, up to complex bidirectional and recurrent multi-state structures that take into account the recurring start and stop of medication. We also investigate modelling choices, such as choosing a time-scale for the transition rates and borrowing information across transitions.

**Results:**

Each structure has its own utility and answers a specific research question. However, the more complex structures (bidirectional, recurrent) enable accounting for the intermittent nature of prescribed medication data. These structures deliver estimates of the probability of being under medication and total time spent under medication over the follow-up period. Sensitivity analyses over different definitions of the medication cycle and different choices of timescale when modelling the transition intensity rates show that the estimates of total probabilities of being in a medication cycle over follow-up derived from the complex structures are quite stable.

**Conclusions:**

Each research question requires the definition of an appropriate multi-state structure, with more composite ones requiring such an increase in the complexity of the multi-state structure. When a research question is related with an outcome of interest that repeatedly changes over time, such as the medication status based on prescribed medication, the use of novel multi-state models of adequate complexity coupled with sensible modelling choices can successfully address composite, more realistic research questions.

**Supplementary Information:**

The online version contains supplementary material available at 10.1186/s12874-023-01905-9.

## Introduction

Multi-state models are used in a variety of epidemiological settings, enabling the study of individuals transitioning through different states across time, portraying with sufficient complexity the real-world issue under study and providing useful and meaningful predictions [1–11]. Measures that can be estimated via the use of multi-state models include, but are not limited to, the probability of being in a state (or a cluster of states) across time, the probability of transitioning from one state to another, the mean length of stay in a state, and the probability of ever visiting a state, as well as the hazard rates/ratios for each transition. Typical examples of multi-state models applications are studying acute [10] or chronic disease progression [4, 7], recurrent events such as repeated hospitalizations [6] and cost-effectiveness in health economic settings [11]. In each setting, the multi-state structure used depends on the research question of interest and the information available in the research data. If a simple measure such as the probability of an event is of interest, then a single event, simple survival analysis may suffice. The information available in the data also drives which kind of research questions can be explored. When the data include information about a state of interest repeatedly over time, more complicated multi-state structures can be implemented, making use of the full richness of the data, to answer composite, realistic research questions. Accompanying the application of more complex multi-state structures, a series of choices with regard to modelling the transition rates, such as choice of timescale and sharing information across transitions, are also available.

Following a diagnosis of breast cancer (BC), many women experience psychological distress including feelings of sadness, fear, anxiety, and depression. The association between BC diagnosis and the development of depression has been previously studied, either using logistic regression [12] or survival analysis for time to first depression diagnosis [13], with an increased risk of depression for individuals diagnosed with BC or cancer in general, compared to a matched population comparison group. As routine primary care information on a diagnosis of depression is often unavailable, many researchers turn to administrative drug prescription databases in order to study proxy measures of mental health on a population level [14–16]. Prescription data offers a readily available, affordable, quantifiable, population-wide measure of antidepressant drug use which can be useful as a proxy of quality of life, including psychological status over time [17].

Breast Cancer Data Base Sweden 2.0 (BCBaSe 2.0) is a linked research database that includes data on dispensed drug prescription from the Swedish Prescribed Drug Registry both before and after a diagnosis of BC, repeatedly over time, from 2006 to 2013, with this information also being available for an age-matched population comparison group of BC free women [18]. The aim of the study is to explore different and novel research questions using the registry-based repeated prescriptions of antidepressants, building from simple ones to more complex, realistic ones by using multi-state structures ranging from single-event survival analysis up to developing bidirectional and recurrent multi-state structures that account for the recurring start and stop of medication.

Based on the longitudinal nature of the prescribed antidepressants data and under certain assumptions, we can classify each woman as being on a medication cycle or a discontinuation period, a status that can change multiple times during follow-up. We start from simpler and commonly used structures such as a single-event survival model (two-state structure) studying the risk for antidepressant medication initiation (first medication cycle), move to a competing risk setting with antidepressants initiation and death as competing events and then a three-state illness-death model, allowing an individual to transition from medication initiation to the death state. We then add a medication discontinuation period state, allowing the study of being in the first antidepressant medication cycle. A backwards transition from a medication discontinuation period state back to a new medication cycle is added (bidirectional structure), allowing an individual to be able to transition multiple times between a period of medication use and a discontinuation period. Then a multi-state structure with recurrent couples of medication cycles and discontinuation periods is proposed, allowing for a more flexible modelling of the medication use patterns, with or without sharing information across transitions.

The bidirectional structure along with the recurrent multi-state structure enabled us to study, among others, the total probability of being and total length of stay in a medication cycle on a population level or conditional on entering a medication cycle, making use of the full richness of information found in the prescribed drug register. The simpler structures do not make full use of the richness of the data, answering simpler, yet still important research questions. We also advocate sensitivity analyses to investigate how probability estimates derived from the more complex structures, are influenced by different medication cycle definitions or different timescale approaches when modelling the transition intensity rates.

## Methods

### Multi-state structures

**Fig. 1:**
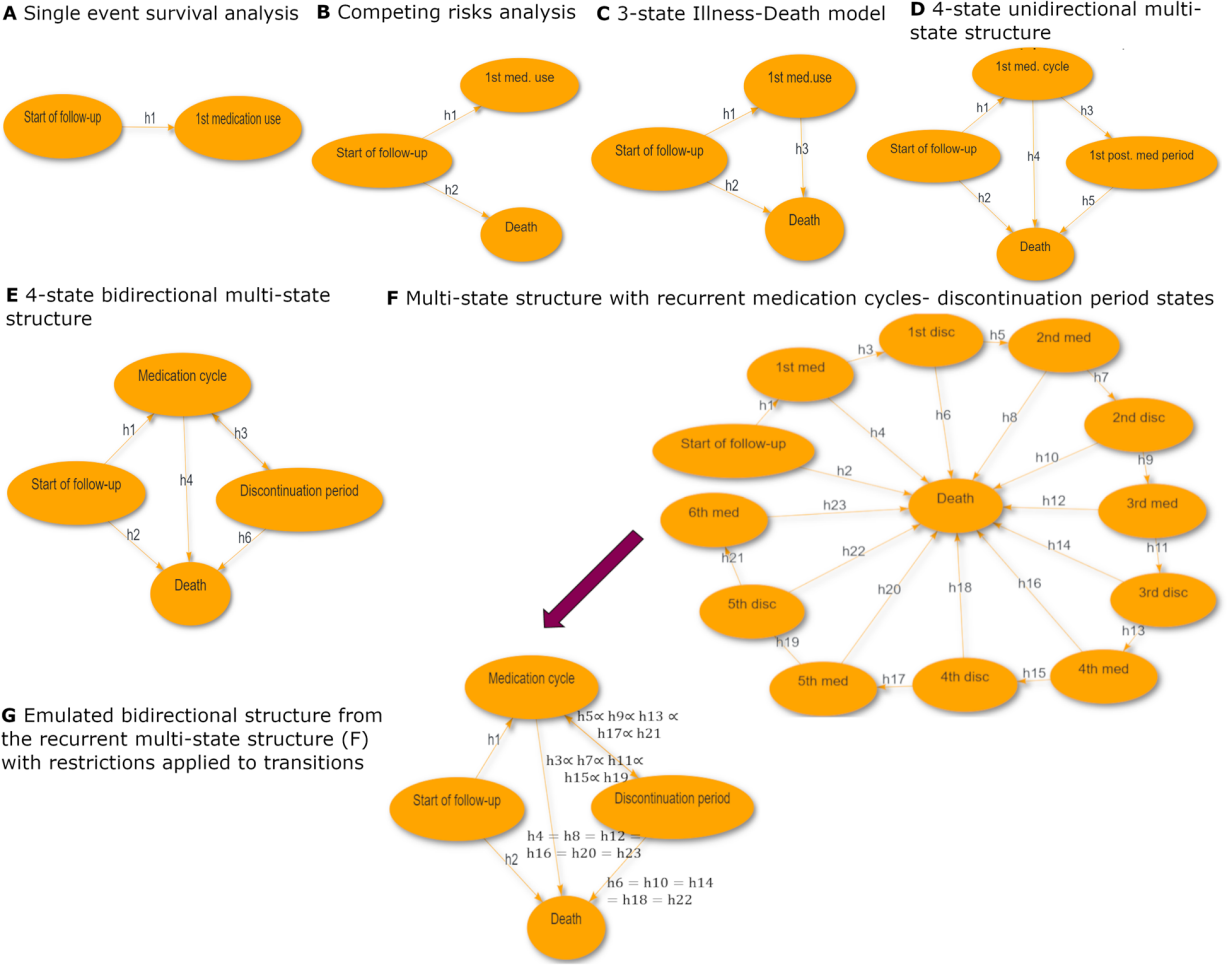
Multi-state structures overview. **A** Single-event survival analysis, **B** Competing risks, **C** 3 –state Illness-Death model, **D** Multi-state model with medication discontinuation state (4-state Unidirectional model), **E** Bidirectional structure between medication cycles and discontinuation periods (4-state Bidirectional model), **F** MSM structure with recurrent medication cycles and discontinuation periods, **G** Recurrent MSM structure with restrictions—“Emulated bidirectional” structure

Figure 1 provides an overview of the multi-state structures used in the current study, from the simplest structural approach (Fig. 1A Single-event survival analysis) to the most complex model (Fig. 1G: MSM with recurrent medication cycles/medication discontinuation periods with restrictions applied). The graphs presented in Fig. 1 were produced via the interactive web-tool MSMplus [19]. The traits, results, interpretation, advantages and drawbacks of each structure are presented in the results section.

Table 1 corresponds each multi-state structure with a specific research question in terms of interpretation of probabilities. In addition to probabilities, other measures of interest can be derived and presented such as transition intensity rates and ratios as well as restricted expected length of stay (or length of stay for short) in each state, probability of ever visiting a state and many more, each with a different interpretation depending on the multi-state structure used. For example, in a 3-state Illness-Death model, the length of stay in the medication use state can be interpreted as the life expectancy of an individual after their antidepressant medication initiation. Transition intensity rate ratios and restricted expected length of stay measures as derived from the different multi-state approaches are presented and interpreted in Additional file 1 (Figures A2 and A3). The main focus of this study is on the medication cycle and discontinuation period states. Therefore, we discuss and interpret the estimated measures of each multi-state structure in the context of those two states.

**Table 1:** Interpretation of probabilities estimated from each multi-state structure

Multi-state structure	Research questions answered in terms of probabilities
Single-event survival analysis of time to antidepressant medication initiation (Fig. 1A)	What is the probability of ever been prescribed medication in the hypothetical situation that the individual cannot die due to any causes?
Competing risks for time to medication initiation with death as a competing event (Fig. 1B)	What is the probability of ever been prescribed medication up to time $t$ after the start of the follow-up, accounting for the fact that individuals may die?
3-state Illness-Death model adding a transition from medication initiation to death (Fig. 1C)	What is the probability of ever been prescribed medication and still be alive up to time $t$ after the start of the follow-up?
4-state unidirectional multi-state model with a medication discontinuation state (Fig. 1D)	What is the probability of being in the **1**^**st**^ medication cycle since start of follow up/ since entering the **1**^**st**^ medication cycle?
4-state Bidirectional multi-state structure with medication discontinuation state (Fig. 1E)	What is the probability of being in **a** medication cycle (or in **a** medication discontinuation period) since the start of follow-up or given entering one?
Recurrent events multi-state structure (with or w/o restrictions) (Fig. 1F and G)	• What is the total probability of being in **a** medication cycle since the start of follow-up or given entering the 1^st^, 2^nd^, 3^rd^ one? • What is probability of being in **the current** medication cycle given entering the 1^st^, 2^nd^, 3^rd^ medication cycle or the 1^st^, 2^nd^, 3^rd^ discontinuation period?

The list above is not an exhaustive list of multi-state structures that can be used and research questions that can be addressed. The estimated measures can be compared between the exposure groups of interest. We highlight in **bold** the subtle differences in phrasing the research questions when interpreting the probabilities of multi-state structures D, E, F and G (1^st^,a, the current medication cycle)

#### Single event survival analysis

If our interest lied in time to first antidepressant medication, a simple hazard model would suffice. In this case, the individual is at risk of transitioning from an initial, medication-free state to the first medication cycle (medication initiation) (Fig. 1A). In this type of analysis, the rate of that transition is the estimate of interest, as well as the relevant risk differences and risk ratios between different groups. In this simple, two-state multi-state model, the derived probability is interpreted as the probability of ever been prescribed medication in the hypothetical situation that the individual cannot die due to any causes. This structure fails to account for the fact that individuals diagnosed with BC have higher risk of dying compared to healthy individuals. Due to this fact, the derived probabilities potentially overestimate the outcome of interest, that is, the probability of ever antidepressant medication initiation up until time $t$. The approach that follows considers the competing risk of death when estimating probabilities of antidepressants initiation.

#### Competing risks

If we use a competing events structure, we allow the individual found in the initial medication-free state to experience either the medication initiation state or the competing event of death (Fig. 1B). This approach takes into account the competing risk of death in the estimation of the probability of experiencing the first antidepressants medication. We can interpret this probability as “the probability of antidepressant medication initiation up to time $t$ after the start of the follow-up”. Even though the competing risks approach considers the competing risk of death, it does not permit the individual to leave the state of experiencing the first medication use. Thus, more complex multi-state structures are in need if our interest lies not only until but also after the first medication use.

#### Three-state illness- death multi-state model

If we allow a transition between the medication initiation and the absorbing state of death, the individual can experience death after medication initiation (Fig. 1C). This structure allows the study of the probability of having experienced the first medication use while also allowing individuals who have entered this state to move to the death state. However, the medication initiation state consists of people who either stayed in a medication cycle for the rest of their follow-up or, more likely, moved on to discontinuation periods or subsequent medication cycles. The 3-state Illness-Death multi-state model, as it is called, is not able to discern this issue and thus is not appropriate for studying the probability of being in a medication cycle or the length of stay in that cycle. A multi-state model that includes a discontinuation period state would allow for the study of being in the first medication cycle.

#### Four-state multi-state model with a discontinuation period state

By adding a discontinuation period-state (Fig. 1D), the individual is now allowed to either go to the absorbing state of death or to a medication discontinuation period after entering the 1^st^ medication cycle state. This addition allows us to shift our focus from the probability of ever been prescribed medication to “the probability of being in the 1^st^ medication cycle” at time $t$ since the start of follow-up or time since entering the 1^st^ medication cycle. Under this structure, the estimated probability for the 1^st^ medication discontinuation period can be interpreted as “the probability of ever having exited the 1^st^ medication cycle and still be alive up to time $t$”, consisting of people who either stayed in a medication discontinuation state for the rest of their follow-up or experienced additional medication cycles later on during follow-up. Thus, if probability of being in the 1^st^ medication discontinuation period and subsequent medication cycles and medication discontinuation periods is of interest, we need to allow an individual to be able to leave the 1^st^ discontinuation period and enter in a new medication cycle.

#### Four-state bidirectional model

A bidirectional multi-state structure can be built by allowing the individual to re-enter a medication cycle after entering a medication discontinuation period (Fig. 1E). Therefore, a back-transition is inserted from the discontinuation period state to the medication cycle state, allowing the individual to move back and forth multiple times -without a limit- among these states. The existence of the fourth state (discontinuation period) and the double transition arrow between this state and the medication cycle state serves a certain purpose. Allowing for a medication-free state that is different from the initial “Start of follow up” state and putting a back-transition towards a medication cycle allows the transition rate for a next medication cycle to differ from the transition rate from the starting state to the first medication cycle. Despite its simple application and interpretation, this structure imposes the same transition rates between a medication cycle and a discontinuation period (and vice-versa) irrespectively of the number of previous medication cycles (or discontinuation periods). While a time-varying covariate of previous medication cycles could be added in the transition rate models, the estimation of measures other than the transition rates and ratios is not possible under the Markov or the semi-Markov assumption. This means that an individual has the same transition rate from a medication cycle to a discontinuation period (and vice-versa) no matter how many previous cycles they may have experienced, an assumption that is not very realistic in our setting. A recurrent multi-state structure allows for the separate modelling of a transition rate between each subsequent couple of medication cycle and discontinuation period (and vice-versa) thus accounting for past transitions via its own structure and is described below.

#### Recurrent multi-state model

A flexible approach when dealing with recurring states is to fit a recurrent multi-state structure that consists of repeated, ordered events/couples of medication cycles- discontinuation periods (Fig. 1F). This structure can be more flexible than the bidirectional model structure, as it allows the separate modelling of each transition rate without imposing same transition rates every time an individual moves from a medication cycle to a discontinuation period (and vice-versa). Due to recurrent states gradually becoming sparsely populated, there is a need for setting a threshold to the number of medication cycles and discontinuation periods to be modelled. In the case of this study, a maximum of six medication cycles are modelled, with subsequent medication cycles and discontinuation periods being ignored. Individuals that enter the 6^th^ medication cycle are characterized as chronic antidepressant medication users and stay in this state until the end of their follow-up or move towards the death state.

By applying the current multi-state structure, we can estimate probabilities with useful clinical interpretation such as “What is the total probability of being in a medication period or a discontinuation period since the start of follow-up (total probability on a population level) or given entering the ${1}^{st}, {2}^{nd},\dots , {K}^{th}$ medication cycle or discontinuation period?”, taking into account past medication cycles via the multi-state structure itself, a trait that the aforementioned bidirectional model lacked. However, using recurrent multi-state structures comes with its own drawbacks. A small to moderate increase in the number of states can greatly increase the structural complexity. Apart from the starting state (start of follow-up) and the absorbing state (death), there will be $K$ medication cycle states (${1}^{st}, {2}^{nd},{3}^{rd},\dots , {K}^{th}$) and $K-1$ post-medication period states (${1}^{st}, {2}^{nd},{3}^{rd},\dots , {K}^{th}-1$), leading to transitions having sparse data as state order progresses. This data sparsity can make transition-specific estimations troublesome, leading to convergence issues and low precision. Keeping the modelling of the covariate effects and the baseline transition rates simple or applying restrictions to the transition rates via shared parameter estimation for certain transitions, can address, at least partially, these issues.

#### Emulated bidirectional structure- recurrent model with restrictions

Under reasonable assumptions, sharing information across transitions of a multi-state structure in order to address the data sparsity issue described above is possible by imposing constraints in the parameter estimation. For the recurrent multi-state structure described (Fig. 1F), it can be assumed that transition rates towards death are not influenced by the number of past medication cycle states. This translates in transition rates from all medication cycle states to death being restricted to be the same and transition rates from all discontinuation period states to death to also be the same. Additionally, it can be assumed that each new transition between medication cycle and discontinuation period (and vice-versa) has a common underlying relationship with the time to experiencing the transition event (same shape) but on different scale (proportional). This translates to imposing commonly shaped, proportional transition rates from medication cycles to discontinuation periods and vice-versa. This group of assumptions/ restrictions conceptually simplify the recurrent multi-state structure back to a bidirectional-like structure which we refer to as “Emulated bidirectional” (Fig. 1G). Contrary to the previous structure (Fig. 1F) where the parameters of each transition were estimated separately, the joint parameter estimation among multiple transitions of the current structure (Fig. 1G) is computationally challenging and leads to excessive memory usage. Due to limitations in the maximum memory allocation (150 GB), this structure was fit to a sub-sample of the study, including all the cases and randomly choosing 2 BC- free individuals per BC case (1:2), while time-dependent effects of the case variable were allowed only for the transition from the starting state (start of follow-up) to the 1^st^ medication cycle and death. The advantages of emulating a bidirectional structure by applying restrictions to the initial recurrent structure is that we can tackle the issue of sparse data in high order states, gain precision in the estimations and conceptually simplify the multi-state structure back to a bidirectional-like structure, while allowing for different transition rates between each couple of medication cycle and discontinuation period states (and vice-versa). However, the “Emulated bidirectional” structure carries some of the drawbacks of the unrestricted recurrent multi-state model, making the assumption that individuals that enter the 6^th^ medication cycle are characterized as chronic antidepressant medication users, while the initial bidirectional structure is free of this limitation. Additionally, due to its excessive memory usage, there are limitations as to how flexibly we can model the baseline transition rates and the covariate effects.

### Modelling the transition rates

Each transition-specific hazard (transition intensity rate) is estimated as a function of time via the use of FPSM [20] (Model expressions provided in Additional file 1) via the merlin package in Stata [21]. In all transitions, the baseline transition intensity rate is modelled with restricted cubic splines with four degrees of freedom ($df=4$). The timescale used for all transitions will be time since entering each state, known as semi-Markov or clock reset approach. As we aim to explain the main concepts of different multi-state structures, we limit to simple modelling of the age at diagnosis/start of follow-up, with the main covariate of interest being the diagnosis of BC. For all transitions, the effect of age at diagnosis/ start of follow-up is included with main effects as a categorical variable with four groups, namely " < 50 years old", "50–59 years old", "60–69 years old", "70 years old plus". The main covariate of interest is the group that each individual belongs according to diagnosis (“BC free individual”, “BC diagnosis”) and is included in the models as a categorical covariate (case status). Time-dependent effects of the case status variable (non-proportional hazards) are allowed in the model via the use of restricted cubic splines with 3 $df$ for all transitions towards a medication discontinuation state and the transition from start of follow-up towards death for all structures except the “Emulated Bidirectional” one due to memory usage limitations. Interaction terms between the age groups and the case status covariate were also included in the models. All predictions will refer to the age group 60–69 years of age at baseline. To derive the measures of interest, individuals of a specific covariate pattern are simulated based on the transition-specific FPSMs and the matrix of possible transitions via Stata *predictms* command, a simulation-based approach of time to events. For more details, see the study from Crowther and Lambert [22].

### Timescale approaches

For multi-state structures, similarly with simpler survival settings, the choice of timescale for the transition rates is an additional factor that should be taken into consideration [23]. Under a clock forward approach (Markov assumption) all the transitions of a multi-state structure have a common timescale $t$ which refers to the time since diagnosis/start of follow-up when modelling the transition intensity rates among the different states and the probability for a future state depends only on the current state. Under the clock reset approach (also semi-Markov or Markov renewal model), the transition intensity rate between two states is a function of the time $t$ since entering the current state [5, 9]. A mix of these two approaches can also be used (clock mix), if, based on subject matter knowledge, certain transition rates are more likely to be functions of time since the start of the follow-up while it is more natural for others to be functions of time since entering the current state. In the present setting, transitions rates towards death could be modeled with time $t$ since the start of follow-up as the timescale (clock forward) while transitions to non-death states (medication cycles and discontinuation periods) could be modeled with time $t$ since entering the current state as timescale (clock reset approach) arguing that it is more natural for these rates to be functions of time since entering the current state (e.g. time since entering current medication cycle). While the clock mix approach may be a more natural choice for modelling the transitions, we chose to use the clock reset approach for the main analyses. Predictions derived from the clock forward and clock mix approaches that are conditional on a state other than the starting state of the process, should be also conditional on a left truncation time $s$ greater than 0, as in the current setting, all individuals start from the same initial state (Start of follow-up) at time 0. On the other hand, under the clock reset approach with time of left truncation $s$ equal to time $r$ of entering the state we want to condition on, the predictions are not dependent on $r$ itself. This means that under the clock reset approach the predicted probabilities can also be reported on time since entering each conditional state, for example, time since entering the ${k}^{th}$ medication cycle, which is more relevant to the research questions posed. Predictions based on the clock forward and the clock mixed approaches were also derived via a sensitivity analysis and can be found in Additional file 1 (Figure A4).

### Study sample, inclusion criteria, definition of medication cycles

Breast Cancer Data Base Sweden 2.0 (BCBaSe 2.0) was created to provide a register-based research resource with data on an unselected cohort of women (and men) with BC in Sweden [18]. The BCBaSe 2.0 database includes information on individuals diagnosed with BC between 1992 and 2012 (*n* = 68,450) identified in three Swedish Regional Clinical Breast Cancer Registers [24] and age and sex-matched individuals as a population comparison group (ratio 1:5) without a history of breast cancer (*n* = 343200) at the end of the year of diagnosis of the index case, with the individuals of the comparison group living in the same county as the case. The mean age at inclusion was 61.8 years (range 19–102) and the cohort has been followed up until 31 December 2013. Loss to follow-up due to migration is taken account by censoring the individual at the date of emigration. By means of record linkages to national demographic and health care population-based registers, information on dispensed prescribed drugs is available. The Prescribed Drug Register was initiated on July 1^st^, 2005, with unique patient identifiers for all dispensed prescriptions in Sweden. The register includes, among other information, dispensed item (substance, brand name, formulation and package), date of prescription and dispensing, dispensed amount, dosage, defined daily doses (DDD).

The initial sample of this study consists of 113296 women from the BCBaSe 2.0 database that have been diagnosed with invasive BC (*n* = 18904) and their healthy-matched population comparators (*n* = 94983), with diagnosis date (or start of follow-up for the healthy-matched individuals) between July 1, 2006 and December 31^st^, 2012 with no prescribed antidepressants for at least 12 months prior the start of follow-up. Out of the sample, 591 women who were initially BC-free became BC cases during follow-up. These women are censored at their diagnosis date and only their initial cohort period are kept, while the rest of their follow-up period as BC cases plus the corresponding BC-free comparators are dropped, yielding a final sample size of 110769, with 18313 women with invasive BC diagnosis and 92454 cancer-free women. The information on dispensed antidepressants is derived from the Prescribed Drug Register, via the Anatomical Therapeutic Chemical (ATC) classification system, using the code N06A. All individuals were followed from their diagnosis date/ start of follow-up until death, emigration, or 31/12/2013, whichever came first. The study is approved by the Regional Ethical Committee of Karolinska Institutet, Stockholm, Sweden (protocol number: 2013/1272–31/4).

The main measures presented in this study are the probability of being in an antidepressant medication cycle or a medication discontinuation period. Therefore, it is important to define how the follow-up of an individual can be divided into such period intervals. According to previous studies [25–27] using prescription data, an individual is considered to be on medication use during the time interval between two consecutive prescription dates, provided that these dates are less than 3 months apart. We can classify an individual as entering in a new antidepressant medication cycle if the new prescription date is more than 3 months away from any previous prescription date. Consecutive prescription dates that are less than 3 months apart are considered to be part of the same medication cycle. Each medication cycle ends at a date that is equal to the last prescription date that is less than 3 months after the previous prescription date plus the duration of the medication given on that date as estimated by the defined daily dose (DDD) and the number of packs of the drug prescribed. This duration is set to a minimum of 3 months and to a maximum equal to the distance of the last prescription date of the current medication period and the first prescription date of the next medication cycle minus 1 day, in order to keep the definition of the medication cycles consistent.

A non-medication period is derived as complementary to the medication cycles. If an individual is not in a medication cycle, then they are by default in a non-medication period. The non-medication period before the first prescription date (antidepressants initiation) and after the start of follow-up can be called the pre-initiation period (Start of follow-up state). All other non-medication periods are essentially defined as medication discontinuation periods. The 3-month distance used for the definition of the medication periods is based on the so-called 90-day rule in Sweden [25]. However, there is no absolute threshold based on which medication periods can be defined. In Sect. 3.2 we perform a sensitivity analysis, presenting results of the multi-state structures based on a 4-month and a 5-month distance between prescription dates when defining the medication cycles.

## Results

Table 2 shows the number and percentage of individuals with or without prescribed antidepressant medication in regards with the date of diagnosis/ starting date of follow-up (Table 2A). The descriptives of Table 2B and C greatly depend on the definition of the medication cycle, for example, the median duration of the first medication cycle is 3 months, equal to the minimum stay in a medication cycle under the 90-day rule used to define the cycles.

**Table 2:** Descriptive statistics for prescribed antidepressant medication. Prescribed antidepressant medication use for BC cases, BC- free population comparators and both, in relation to the start of follow-up, for individuals with start of follow-up on or after 01/07/2006 (A), Distribution of number of antidepressant medication cycles across individuals among those with at least one prescription (B), Median duration of the first five medication cycles and discontinuation periods (C). Prescribed Drug Register data available from 01/07/2007 onwards

A		BC- free individuals		BC cases			Both	
		N (%)		N (%)			N (%)	
Women with no prescriptions within a year prior to or after the start of follow-up)		76,413 (82.7)		14,051(76.7)			90,464(81.7)	
Women with prescriptions more than a year prior the start of follow-up only		5199(5.6)		902(4.9)			6101(5.5)	
Women with prescriptions only after the start of the follow-up		8338(9)		2680(14.6)			11,018(9.9)	
Women with prescriptions more than 12 months prior and after the start of follow up		2504(2.7)		680(3.7)			3184(2.9)	
		92,454 (100)		18,313 (100)			110,767 (100)	
B	Number of antidepressant medication cycles after the start of follow up among those with at least one prescription (*N* = 14,202)							
Number of individuals (N, %)	1	2	3		4	5		6
	6 645 46.79%	2 803 19.74%	1653 11.64%		1070 7.53%	752 5.30%		496 3.50%
	7	8	9		10	11		> 11
	310 2.18%	220 1.55%	116 0.82%		79 0.56%	36 0.25%		23 0.15%
C								
	1^st^	2^nd^	3^rd^		4^th^	5^th^		
Median medication cycles duration (years)	0.25 (3 months)	0.27	0.33		0.34	0.4		
Median discontinuation period duration(years)	0.23	0.15	0.11		0.09	0.8		

### Αpplication of the multi-state models

#### Single event survival analysis

**Fig. 2:**
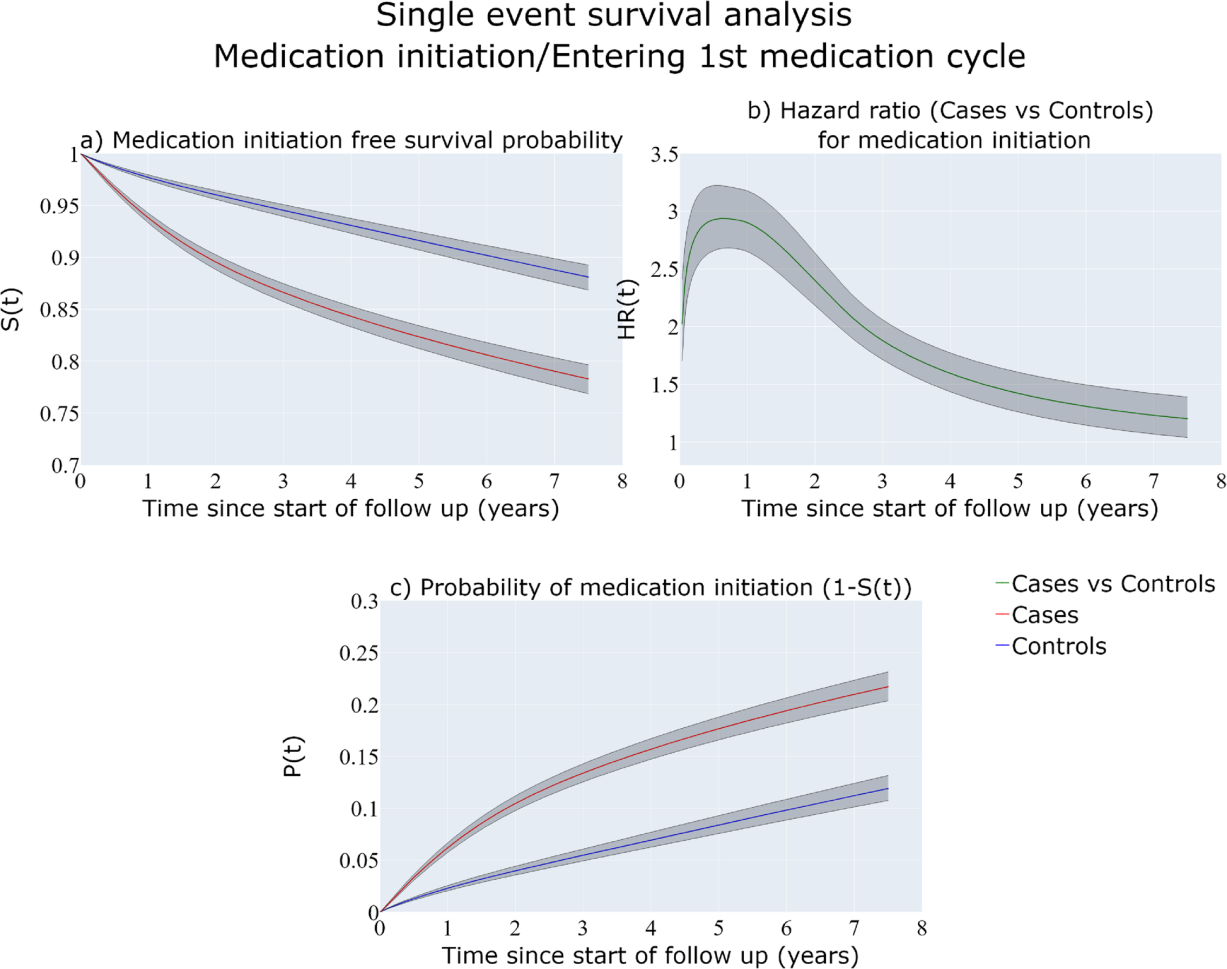
Single-event survival analysis estimates over follow-up time. Medication-free survival probability (**a**), Hazard ratio for antidepressant medication initiation (**b**), probability of antidepressant medication initiation (**c**) for BC-free individuals and cases

Figure 2a shows the estimated survival probabilities (not having medication up until time $t$) for the group of the matched population comparison and the BC cases while Fig. 2c shows the probability of medication initiation up to time $t$ which is $1-S(t)$. The cases have a higher probability for antidepressant medication initiation, with a 20% probability for at least one prescription of antidepressants compared to 10% for the BC-free women within 6 years since start of follow-up. These are the probabilities of ever been prescribed medication in the hypothetical situation that the individual cannot die due to any causes and thus tend to overestimate the true probabilities of ever been prescribed medication. Figure 2b show that BC cases have almost 3 times higher hazard rate for first medication use at 1 year since start of follow-up compared to the BC-free individuals.

#### Competing risks

**Fig. 3:**
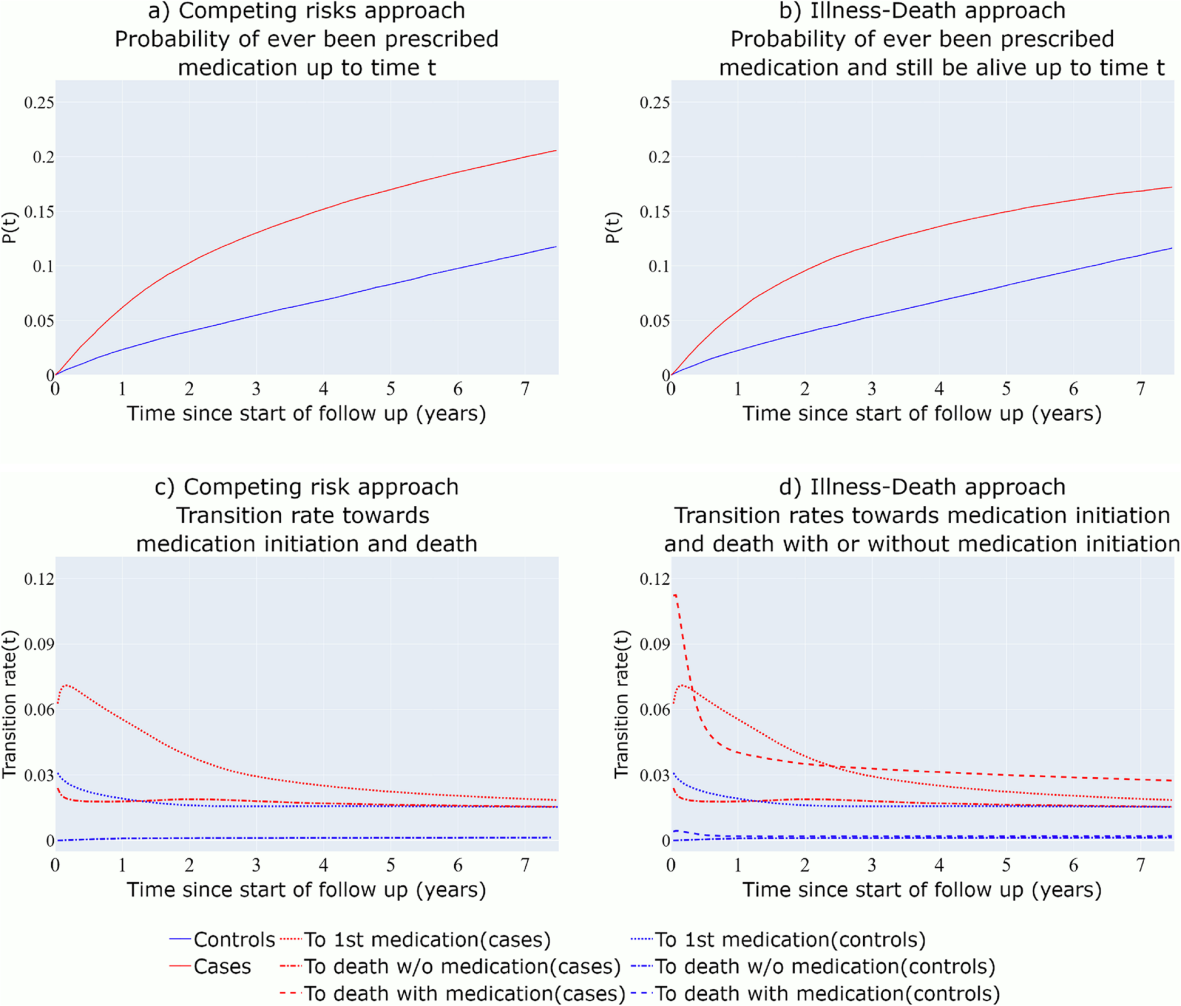
Competing risks approach and 3-state Illness-Death model derived probabilities and transition intensity rates. The hazard rates towards death for the BC-free individuals (with or without medication) are approximately 0.001

In Fig. 3a, the probability of ever been prescribed medication (medication initiation) is derived for the BC cases and the BC-free individuals, having accounted for the fact that the rate of death is much higher for the women diagnosed with BC (red versus blue dash-dot lines of Fig. 3c). It can be observed that in this setting, the estimates from the single-event survival analysis are very close to the ones derived by the competing risks approach (Fig. 2c versus Fig. 3a), as death due to BC does not seem to be a strong competing event for time until antidepressant medication initiation.

#### Three-state illness-death multi-state model

The derived probability in Fig. 3b can be interpreted as the probability of ever been prescribed medication and still be alive up to time $t$ after the start of the follow-up. For the population comparison group, the transition rate from medication initiation towards death is low (Fig. 3d) so the probability estimates of Fig. 3b are very close to those of Fig. 3a (Competing risks structure). However, for women diagnosed with BC, the transition rate towards death both before and after the first medication use is higher (Fig. 3d). For this reason, the estimated probabilities of experiencing the first medication use and still being alive for BC cases are lower than the probability of experiencing the first medication use derived from the competing risks approach.

#### Four-state multi-state model with a discontinuation period state

**Fig. 4:**
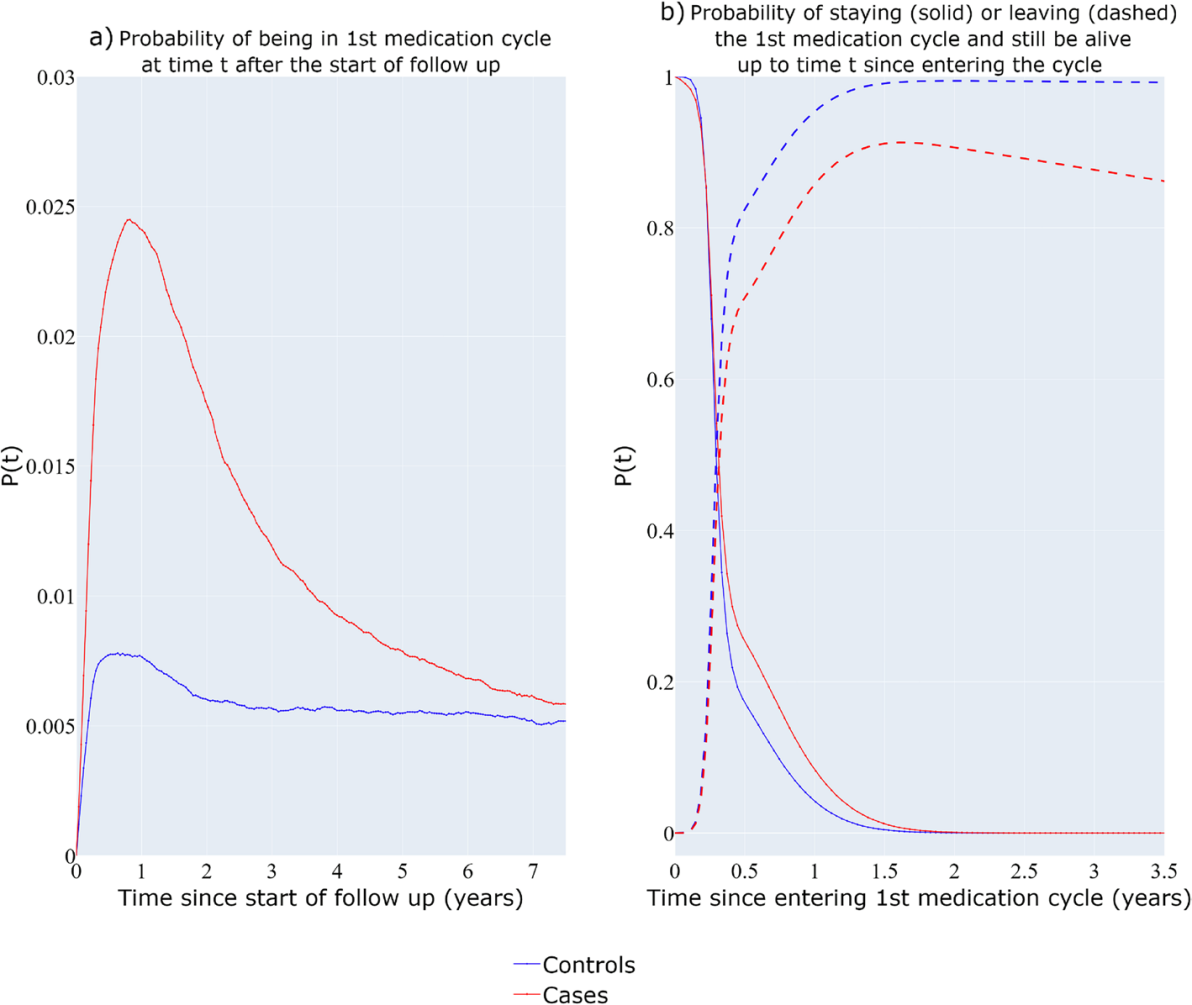
Four-state unidirectional multi-state model with post-medication period state. Probability of being in first medication cycle since the start of the follow-up (**a**), Probability of staying (solid) in the first medication cycle and entering the 1st discontinuation period (dash) for time t since entering the cycle (**b**)

Figure 4a depicts the probability of being in the 1^st^ medication cycle as a function of time $t$ since the start of follow-up while Fig. 4b shows the probability of being in the 1^st^ medication cycle and the probability of heading towards the 1^st^ medication discontinuation period and still be alive, as a function of time since entering the cycle. The overall probability of being in the 1^st^ medication cycle (Fig. 4a) is higher for the cases (red) compared with the BC-free individuals (blue line). The probability of staying in the 1^st^ medication cycles state is higher for the cases for the first 1.5 years since entering the medication cycle.

#### Four-state bidirectional model

**Fig. 5:**
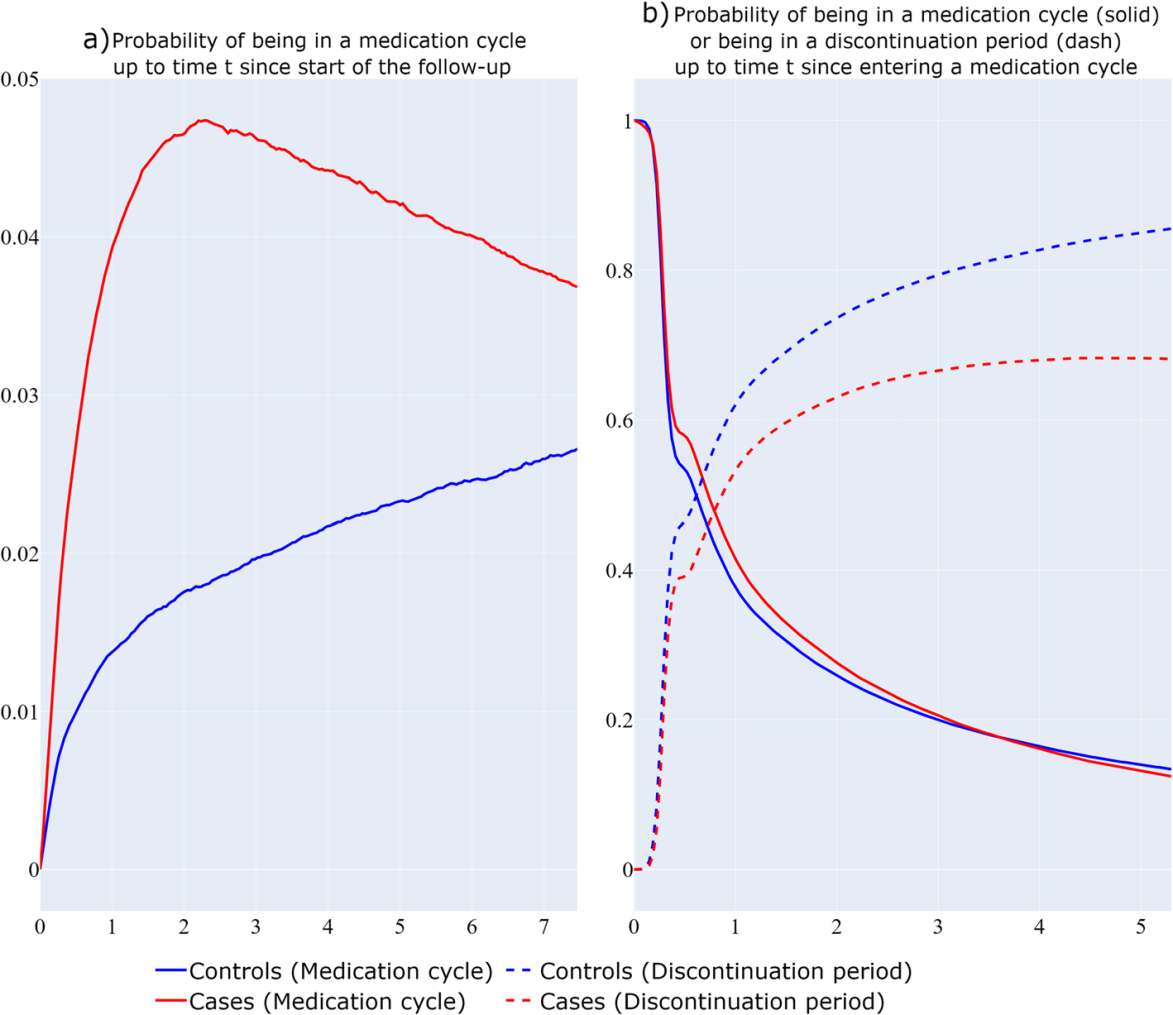
Bidirectional multi-state structure approach. Probability of being in a medication cycle at time t since the start of the follow-up (**a**), Probability of being in a medication cycle (solid) or being in a medication discontinuation period (dash) as a function of time t since entering a medication cycle (**b**)

Figure 5 depicts the probability of being in a medication cycle since the start of the follow-up (5a), and the probability of being in a medication cycle or discontinuation period, given that an individual starts in a medication cycle (Fig. 5b). In Fig. 5a, the BC cases present a higher overall probability of being in a medication cycle compared to the BC-free individuals over the follow-up time. In Fig. 5b it can be observed that BC cases have higher probabilities of being in a medication cycle and lower probabilities of being in a medication discontinuation period compared to BC-free individuals as a function of time since entering a medication cycle.

#### Recurrent multi-state model

**Fig. 6:**
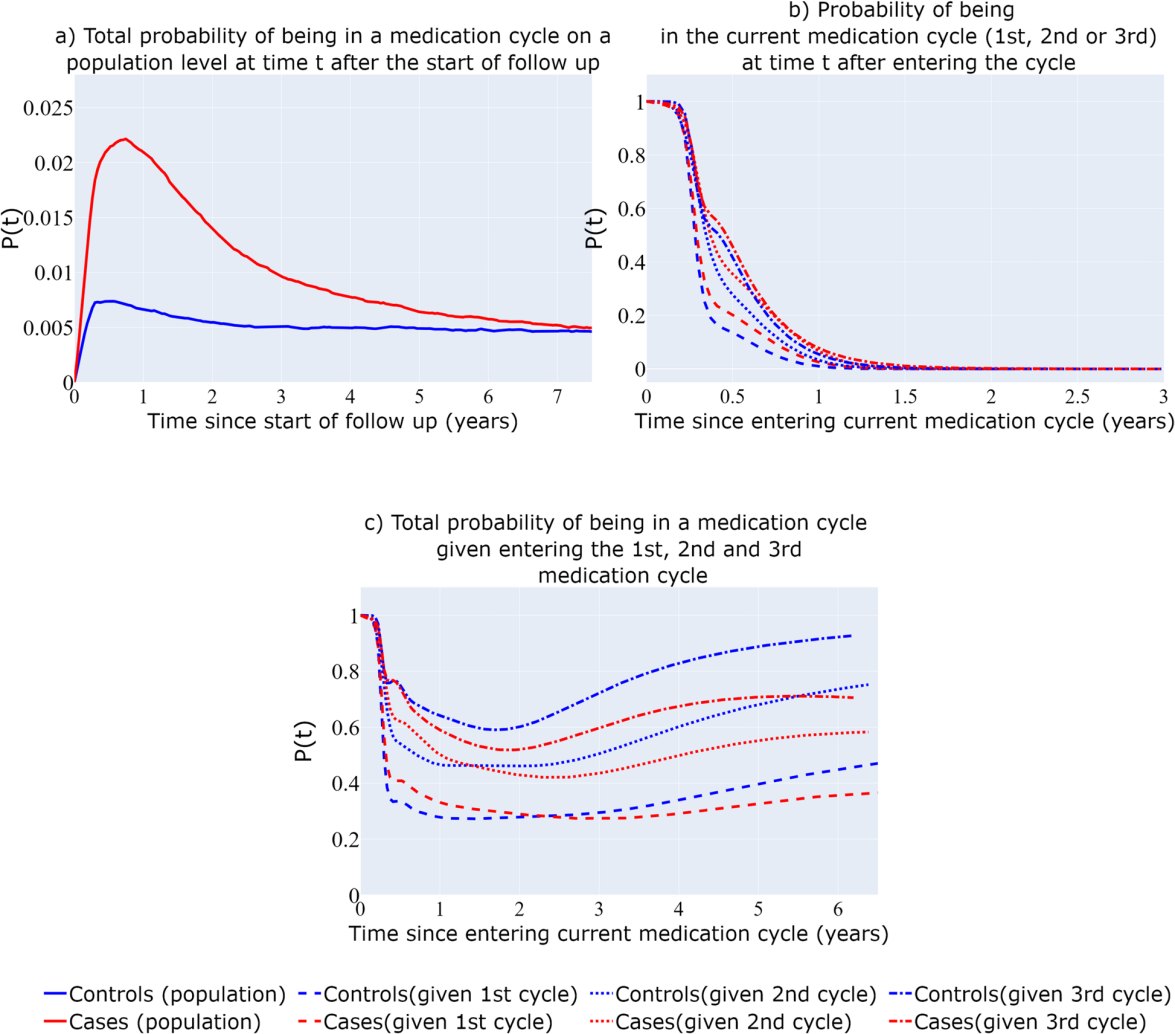
Recurrent events multi-state structure. Total probability on a population level of being in a medication cycle since the start of follow-up (**a**), Probability of staying in current medication cycle since entering it (**b**), Total probability of being in a medication cycle since entering the 1^st^, 2^nd^, 3^rd^ medication cycle (**c**)

Figure 6a depicts the total probability of being in a medication cycle/the prevalence of medication use in the population since the start of follow-up on a population level. While this probability in absolute terms is low, cases appear to have more than three times the probability of being in a medication cycle since their diagnosis compared with the comparison group for the first 2 years of their follow-up. Figure 6b depicts the probabilities for BC cases and BC free individuals of staying in their ${1}^{st}$, ${2}^{nd}$ and ${3}^{rd}$ medication cycle after entering each one of them. The probability of staying in a medication cycle increases with entering each new medication cycle for both BC cases and BC free individuals, with cases having higher probabilities compared to BC free individuals in all the three cycles presented here. Figure 6c presents the total probability of a participant being in a medication cycle (both the current one and all the subsequent ones) as a function of time since entering the ${1}^{st}$, ${2}^{nd}$ and ${3}^{rd}$ medication cycle (Fig. 6c). The total probability of being in a medication period seems to increase as the number of past medication cycles increases, as a function of time since entering a medication cycle. An interesting finding is that, given that a participant experiences her 3^rd^ medication cycle, a BC-free individual has higher total probability of being in a medication cycle compared to a case.

#### Emulated bidirectional structure- recurrent model with restrictions

**Fig. 7:**
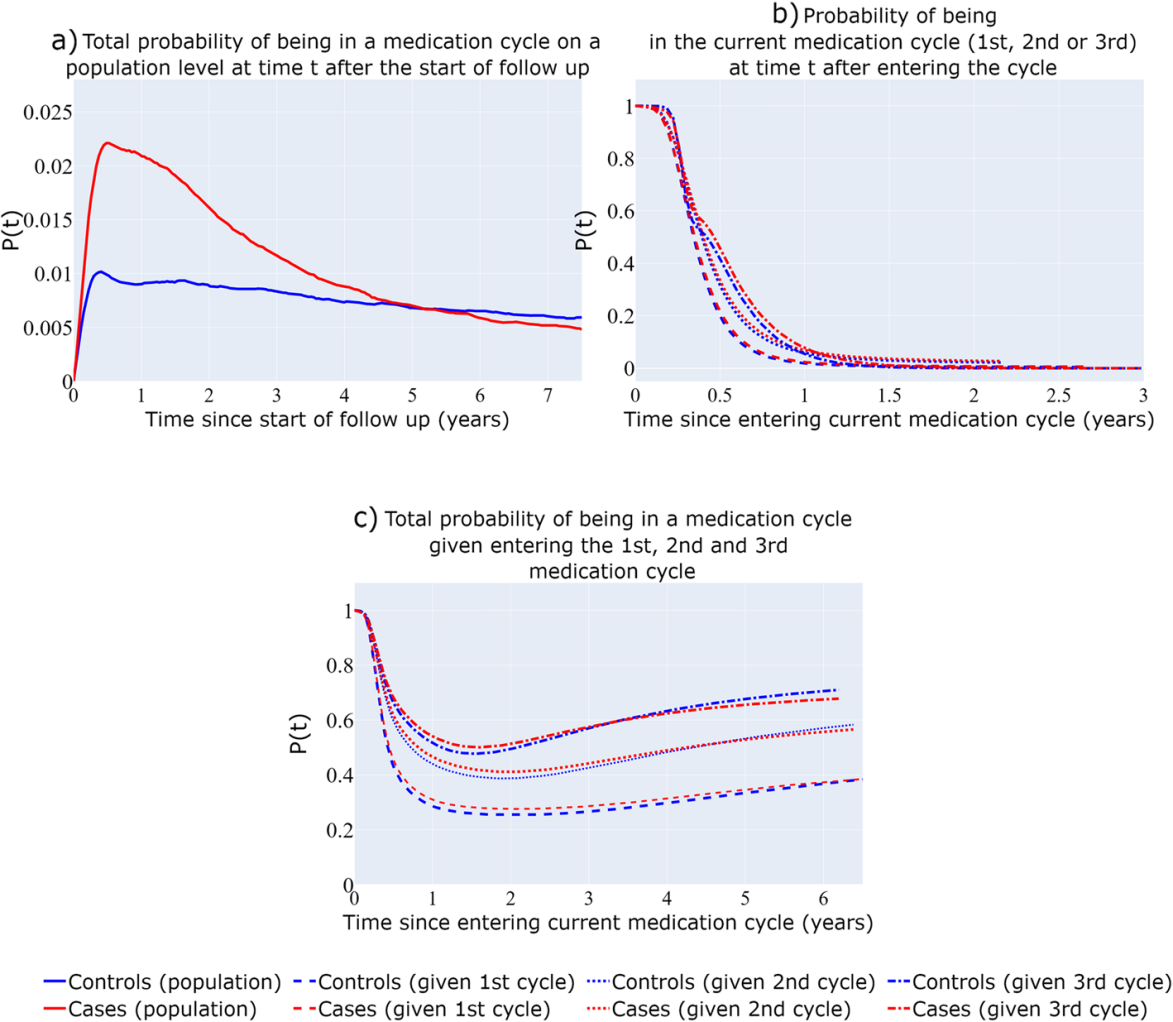
Recurrent events multi-state structure with restrictions (“Emulated bidirectional” structure). Total probability on a population level of being in a medication cycle since the start of follow-up (**a**), Probability of staying in current medication cycle since entering it (**b**), Total probability of being in a medication cycle since entering the 1^st^, 2^nd^, 3^rd^ medication cycle (**c**)

The estimated probabilities of the “Emulated bidirectional” structure (Fig. 1G) depicted in Fig. 7a, b, and c can be interpreted in the same way as those of the recurrent multi-state structure without the restrictions (Fig. 1F), with the estimations of Fig. 6 being similar in shape and scale to those of Fig. 6. It can be observed that, contrary to Fig. 6, the estimated probabilities of Fig. 7 are very similar for BC cases and BC- free individuals (with the exception of Fig. 7a). This is likely because we did not allow for time-dependent effects for the case variable among the transitions from medication cycles to discontinuation periods (and vice-versa), resulting in time-constant transition intensity rates ratios of BC cases versus BC-free individuals that are close to 1 for those transitions (Figure A2b), leading to similar probability estimates of being in the same medication cycle among the two groups.

### Sensitivity analysis- different definitions of medication cycle

**Fig. 8:**
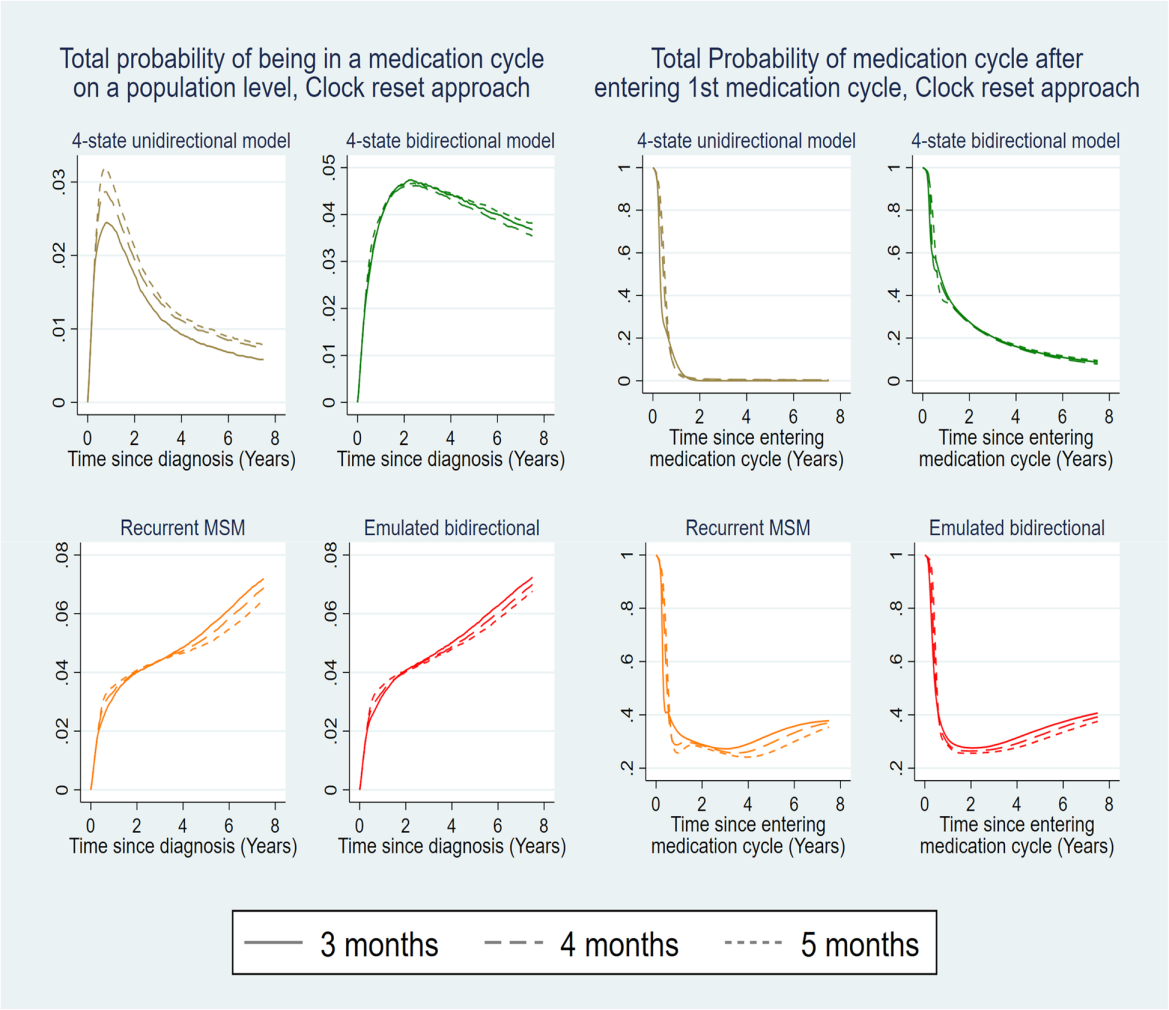
Estimated total probabilities of being in a medication cycle across structures for different medication cycle definitions. Total probability of being in a medication cycle on a population level (left) or given entering the 1st medication cycle (right) for individuals with BC, 50-59 years old at diagnosis, for different definitions of the medication cycles (3 months versus 4 months versus 5 months) for the multistate structures D, E, F and G

In Sect. 2.4, we classified an individual as being in an antidepressant medication cycle from the first prescription date of the cycle (more than 3 months after any previous prescription date) up until the last prescription date of the cycle, plus an extra time period, the maximum between 3 months and the duration of the last prescribed medication treatment of the cycle. It is of interest to assess whether the estimates derived from the multi-state structures are robust to different definitions of the length of a medication cycle. Figure 8 presents probability estimates for the BC cases- probability on a population level of being in a medication cycle and the probability of being in a medication cycle after entering the first medication cycle – under the unidirectional (Fig. 1D) and bidirectional 4-state structure (Fig. 1E), the recurrent multi-state structure (Fig. 1F) and the “Emulated Bidirectional” structure (Fig. 1G). The thresholds of 4 and 5 months are used for the alternative definitions of being in a medication cycle or non-medication period. It can be observed that the probability estimates derived from each multi-state structure are similar under the different definitions of the medication cycles, with the exception of the total probability of being in the 1^st^ medication cycle given by the 4-state unidirectional structure (Fig. 1D) whose estimated probability (being in 1^st^ medication cycle) directly depends on the medication cycle definition. As aforementioned in Sect. 5.4 and Table 2, each structure has a different interpretation of its estimated probabilities, thus not being directly comparable, with the exception of the recurrent multi-state model (Fig. 1F) and the Emulated bidirectional model (Fig. 1G) that have the same underlying structure. Figure A4 of Additional file 1 compares the estimated total probability on a population level of being in a medication cycle across the different multi-state approaches, the different definitions of the medication cycles for the different clock approaches.

## Discussion

We addressed a variety of research questions when dealing with registry-based repeated prescriptions of antidepressants for women with BC diagnosis and BC- free population comparators, using multi-state models, building up from simple towards complex structures, motivating each step of the process. Each step from one multi-state structure to the next one allowed the use of more information available in the prescription data, thus addressing more complex research questions, or added more flexibility in the structure or was motivated in order to address issues/limitations of the preceding structure. While each multi-state structure, no matter how simple, has its own utility and answers specific research questions, we aimed in utilizing the full richness of our data with the more complex structures while also considering different modelling choices.

If, for example, the research question of interest was limited to “What is the probability of ever being prescribed a medication and still be alive up to time t after the start of the follow-up”, then, a three-state illness-death model structure would suffice. However, since we wanted to utilize all the information available in the prescribed drug register regarding antidepressants, more complex research questions such as the probability of being in a medication cycle, including the recurring cycles or studying the susceptibility for antidepressant medication use given past medication cycles can be addressed. Synthetic example datasets with the multi-state data structures used in this study are provided in the Supplementary material. The robustness of the estimated probabilities derived from the different multi-state structures for different definitions of being in an antidepressant medication cycle was assessed via a sensitivity analysis, showing that the complex multi-state structures (Recurrent multi-state, Bidirectional, “Emulated bidirectional”) are relatively insensitive to alternative medication cycle definitions.

As mentioned in the description of the multi-state structures used in this study (Sect. 2), even the more complex models present limitations. The bidirectional model cannot take into account information from past medication cycles to estimate probabilities of being or length of stay in a new medication cycle. Even though we bypass this limitation by applying a recurrent multi-state structure, there is still the issue of the rise in the complexity of the structure, leading to an increased number of states that progressively become sparsely populated, which can cause precision and model convergence issues. In addition, there is the limitation of pooling individuals in a final, recurrence state (6^th^ medication cycle), from which they can only proceed towards the death state, assuming they become, chronic antidepressant users for the rest of their follow-up. Even though the restricted recurrent multi-state model aims to tackle the precision and convergence issues, it still suffers from the chronic antidepressant users assumption as well as the assumptions made regarding the relations between the transition rates which may not necessarily be realistic.

Even though the use of different multi-state structures have been used before on data for demonstrating purposes such as in [2, 9], to our knowledge, this is the first work that addresses probability-related research questions regarding medication use via a series of multi-state models of increasing complexity while also considering multiple modelling choices. Lauseker et al. [7] and Le-Rademacher [28] discuss about different multi-state structures on clinical data about Chronic Myeloid Leukemia (CML) and Acute Myeloid Leukemia (AML) respectively, but they apply a single multi-state structure comparing modelling choices. Meira-Machado et al. [29] evaluate the Markov property of a 3-state Illness-Death model, deriving probability estimates under a semi-Markov and non-Markov assumption and comparing the structure with a single-event survival analysis with the intermediate state as a time-varying covariate, but they do not explore further multi-state structures.

Another issue to consider is choosing the timescale to be used for the transition models of the chosen multi-state structure. In the main analysis we used time since entering current state as the timescale for all transition models (“clock reset”). However, we also implemented the “clock forward” and the “clock mix” [30] approaches as part of a sensitivity analysis in order to explore how robust are the estimations of the complex multi-state structures for different timescale choices when modelling the transition rates (Figure A4 of Additional file 1). The “clock reset” and “clock mix” approach give almost identical estimates, while the “clock forward” approach gives estimates close to the other two approaches. A limitation of this study regarding timescales is that we assumed that each transition rate is a function of either time since the start of follow-up or time since entering the current state. However, it can also be assumed that each transition rate is a function of multiple timescales simultaneously [31]. This modelling assumption can be implemented on a multi-state model framework via the merlin package in Stata [21] or simLexis library in Epi R package [32] and could be compared with the modelling approaches used in the current study in future research.

Other measures derived under a MSM framework may be of primary interest such as the total length of stay in medication cycles over the follow-up time or transition rates and rate ratios for experiencing the next medication cycle among individuals with different profiles (covariate patterns). Due to space limitations, estimation results regarding these measures and their interpretation under each multi-state structure are presented in Additional file 1. A pseudo-dataset is supplied in Additional file 2 which can be used by the code in Additional files 3 and 4 to create the multi-state structures and run the multi-state models discussed in this study.

Finally, since recurrent multi-state models structures are used in this study, it is important to note that recurrent events analyses can also be approached either with recurrent multi-state models with death as an absorbing state or by the joint modelling of recurrent events and the terminal event of death [33–35]. However, since the focus of this study is on exploring different research questions via multi-state models when dealing with registry- based prescription data, expanding on the use of joint frailty models is out of our scope.

## Conclusions

In this study we explored how different research questions can be addressed, ranging from simple to composite ones, surpassing the single-event and competing risks settings, and defining complex bidirectional and recurrent multi-state structures, highlighting the importance of choosing a structure that properly addresses the clinical research question of interest in each case. When information on an outcome of interest that repeatedly changes over time is available, such as the medication status based on prescribed medication, in the presence of other competing events such as death, the use of novel multi-state models of adequate complexity can successfully address composite, more realistic research questions. In addition, during the application of such models, there is a number of modelling choices such as the choice of timescale for each transition and the borrowing of information across transitions that should be explored and evaluated.

## Supplementary Information

**Additional file 1.** Appendix.

**Additional file 2.** Pseudodata.

**Additional file 3.** MSM structures.

**Additional file 4.** MSM analyses.

## Data Availability

The data that support the findings of this study originate from the three Swedish Breast Cancer Quality Registers (from the Stockholm-Gotland, the Uppsala-Örebro, and the Northern regions) and the Prescribed Drug Register. Restrictions apply to the availability of these data, which were used under license for the current study, and so are not publicly available. Details of the application procedure for obtaining the data are available at https://cancercentrum.se/samverkan/cancerdiagnoser/brost/kvalitetsregister/forskning/bcbase-3.0---en-forskningsdatabas-for-epidemiologisk-brostcancerforskning/. A synthetic example dataset is provided in the Supplementary material (“Additional file 2- Pseudodata”) accompanied with code (Additional file 3- Multi-state structures and Additional file 4- Multi-state model analyses) to reenact the multi-state data structures and analyses used in this study.

## Abbreviations

ATC: Anatomical Therapeutic Chemical
AML: Acute Myeloid Leukemia
BCBaSe 2.0: Breast Cancer Data Base Sweden 2.0
BC: Breast Cancer
CML: Chronic Myeloid Leukemia
DDD: Defined Daily Dose
FPSM: Flexible Parametric Survival Models
MSM: Multi-State Models
