# Supplementary material for: Exploring different research questions via complex multi-state models when using registry-based repeated prescriptions of antidepressants in women with breast cancer and a matched population comparison group
Source: BMC Med Res Methodol. 2023 Apr 10;23:87. doi: 10.1186/s12874-023-01905-9 (PMC10084660; doi:10.1186/s12874-023-01905-9)
Supplement: Supplementary file 1 — Additional file 1. Appendix. [file 12874_2023_1905_MOESM1_ESM.pdf]

Appendix

Figure A1 is a schematic representation of the duration of a medication cycle based on the observed prescription dates and the 90-day rule.

Figure A1. Diagram for antidepressant medication cycles. Time gaps of less than three months between two consecutive prescription dates are depicted in deep purple and time gaps of more than three months with pink.

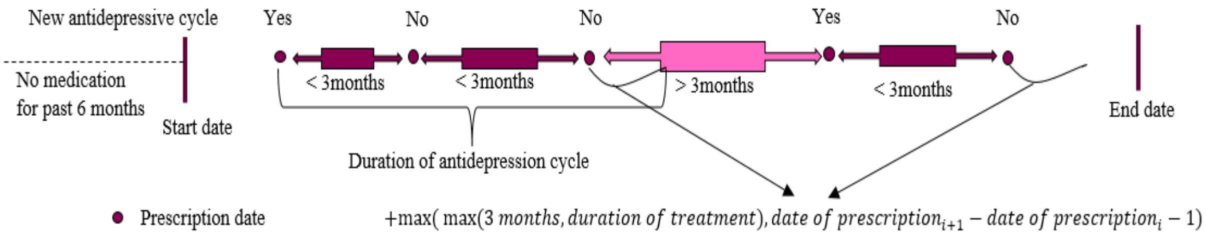

Figure A2. Transition rates ratios for BC cases versus BC-free individuals for the different multi-state structures

a)

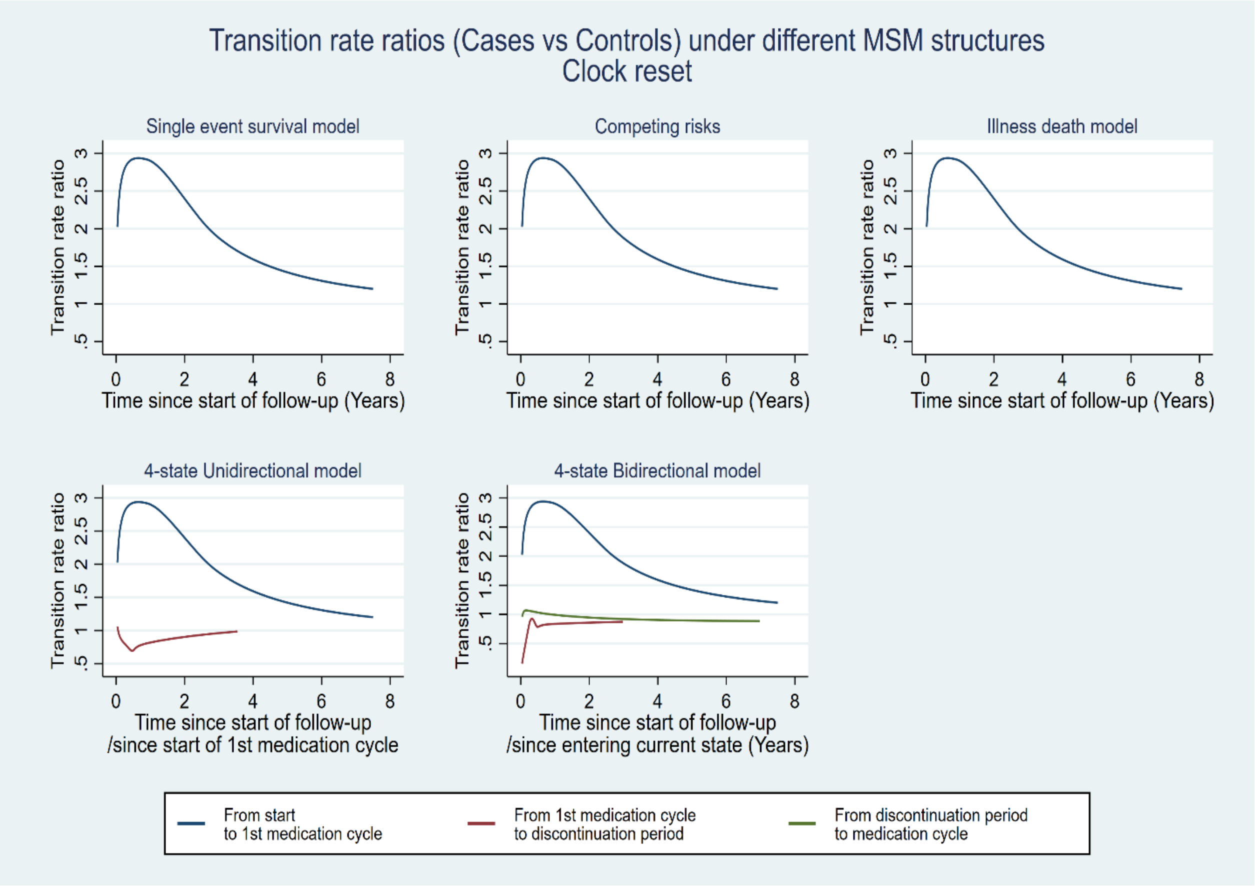

b)

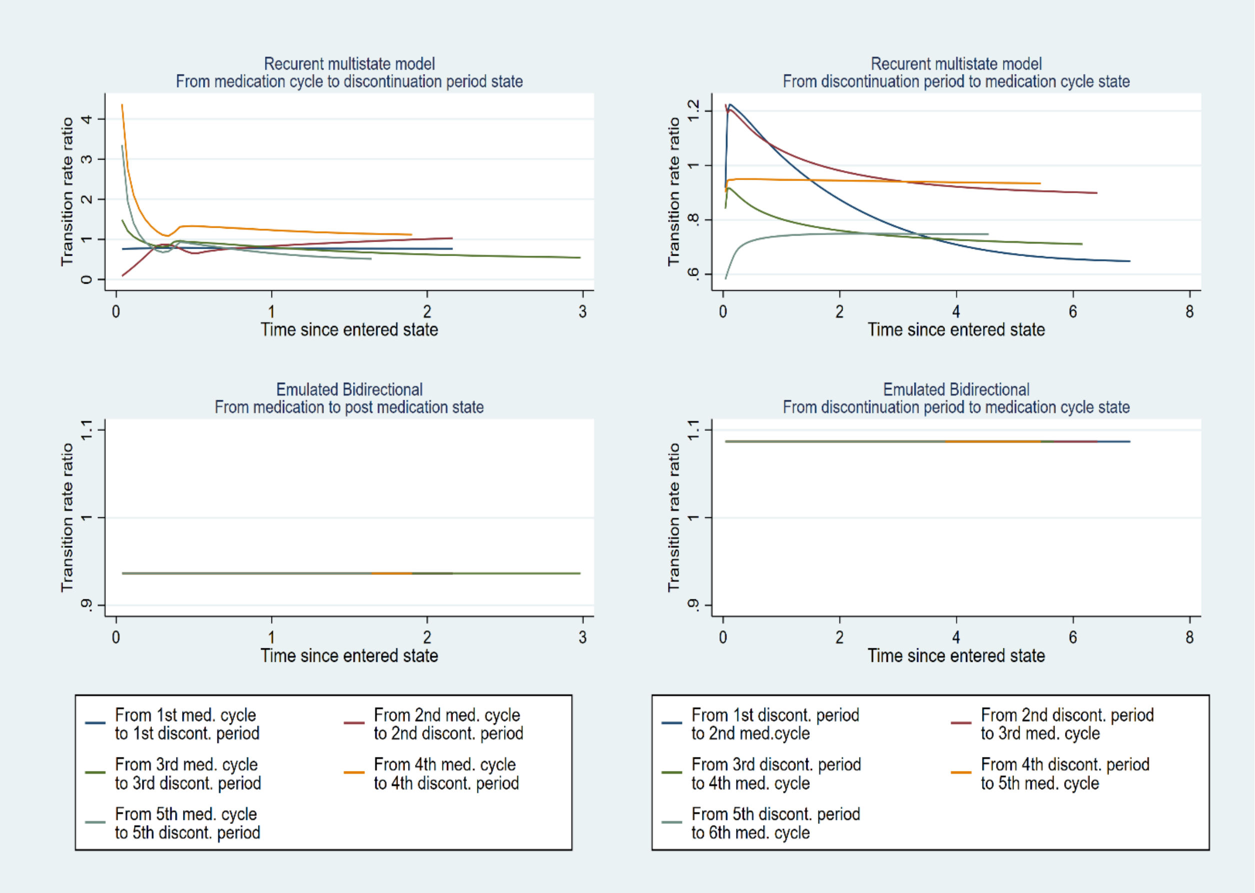

*Transition rate models towards and from medication states under the “Emulated bidirectional” do not allow for time varying effects of the being a case, that is why the transition rate ratios are constant across time. The case variable in our analysis is constrained to have the same effect on the transition rates from medication cycles to medication discontinuation periods (and vice versa). That is why all transitions rate ratios lines of BC cases versus BC-free individuals overlap.

Figure A3. Restricted expected length of stay estimates, with their different interpretations, for BC cases and BC- free individuals among the different multi-state structures.

a)

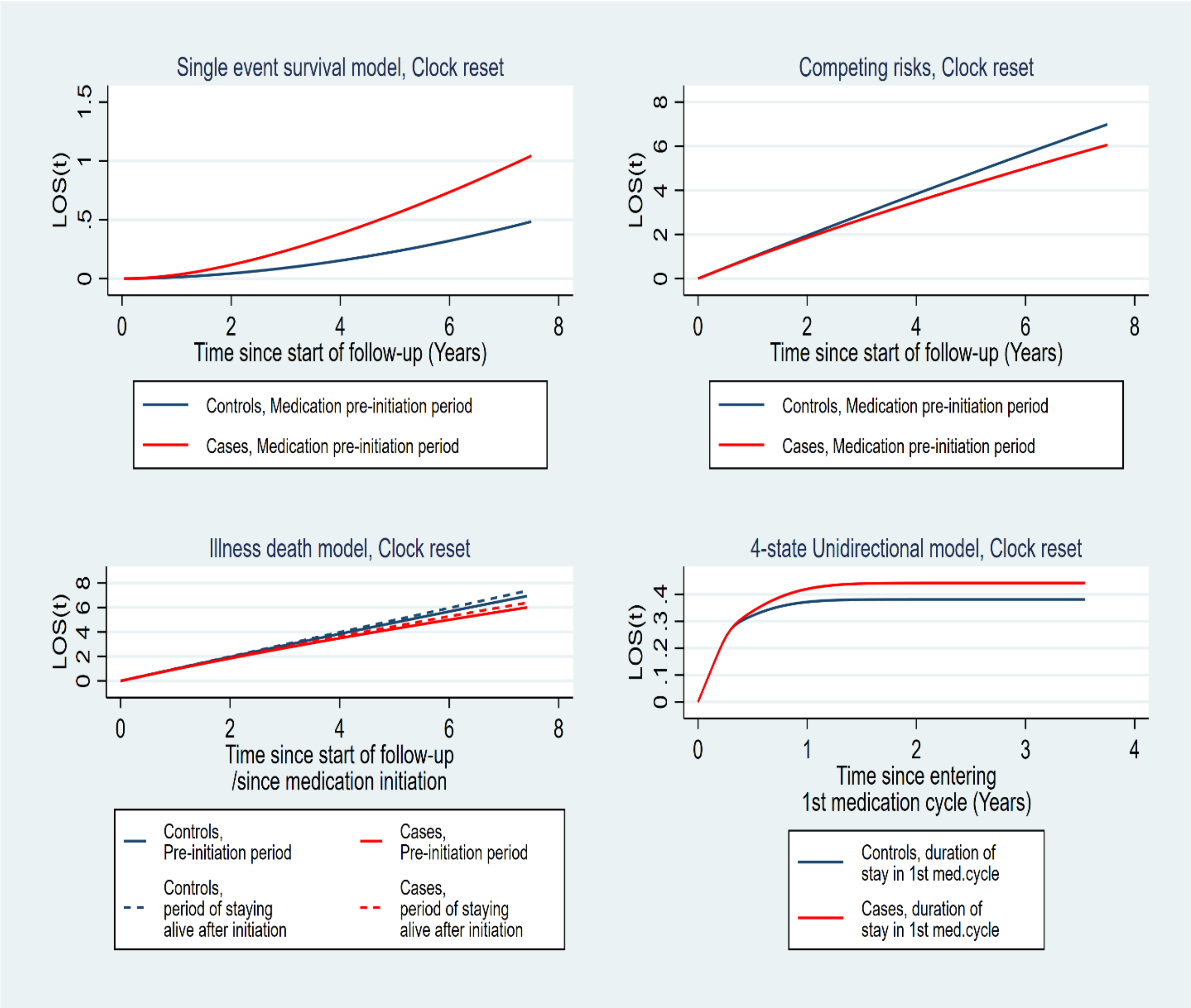

b)

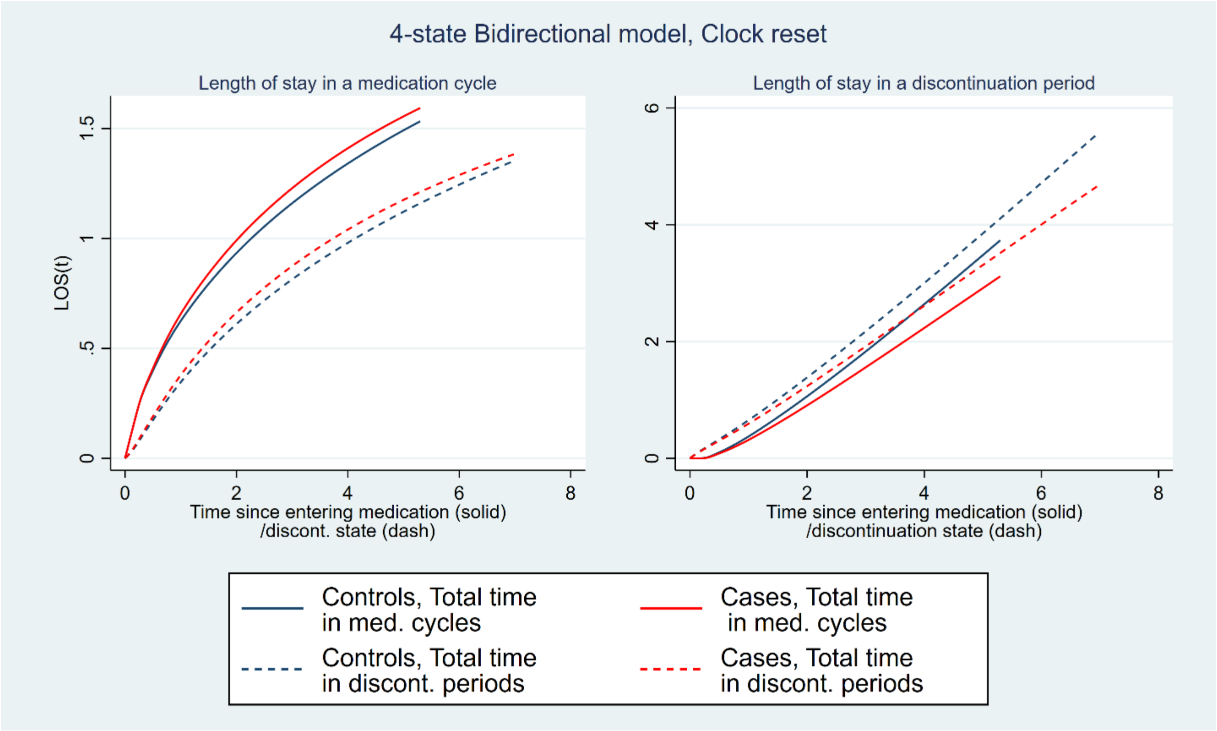

4-state Bidirectional model, Clock reset
Length of stay in a medication cycle
Length of stay in a discontinuation period
LOS(t)
0
.5
1
1.5
0
2
4
6
0
2
4
6
8
Time since entering medication (solid)
/discont. state (dash)
Time since entering medication (solid)
/discontinuation state (dash)
Controls, Total time in med. cycles
Cases, Total time in med. cycles
Controls, Total time in discont. periods
Cases, Total time in discont. periods

c)

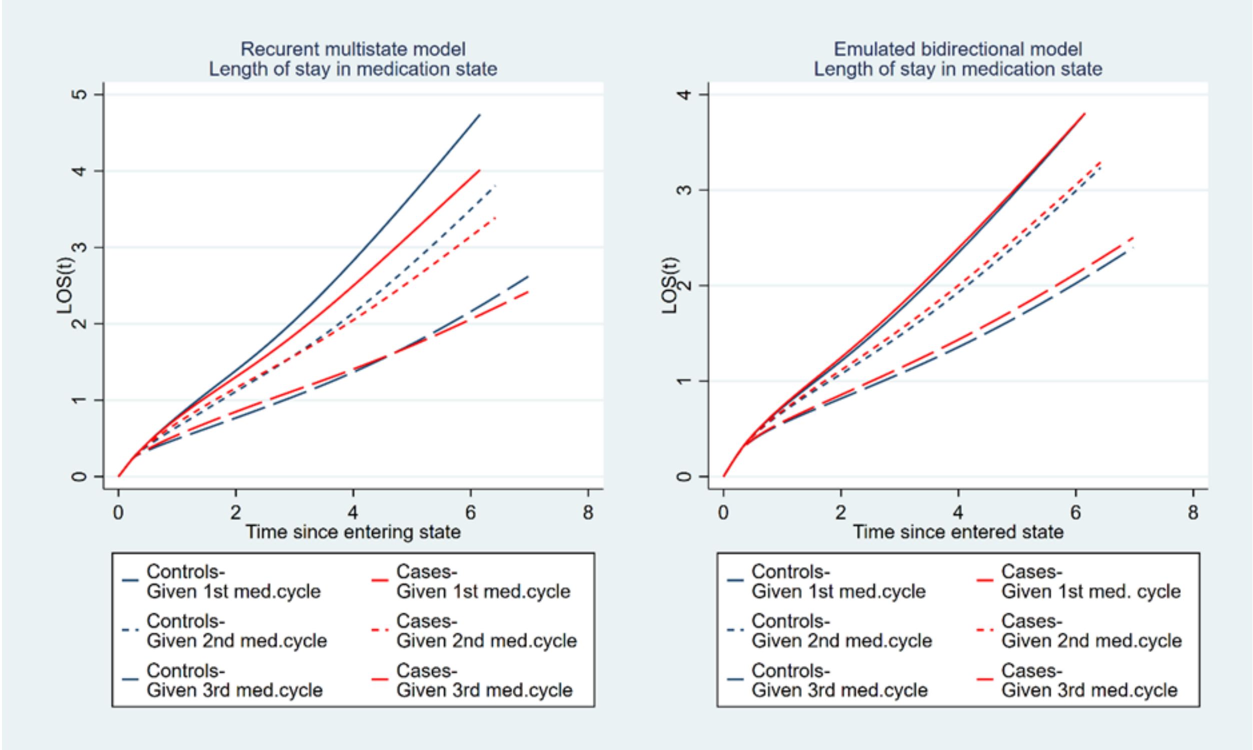

Recurent multistate model
Length of stay in medication state
Emulated bidirectional model
Length of stay in medication state
LOS(t)
0
1
2
3
4
5
0
2
4
6
8
Time since entering state
Time since entered state
Controls-
Given 1st med.cycle
Cases-
Given 1st med.cycle
Controls-
Given 2nd med.cycle
Cases-
Given 2nd med.cycle
Controls-
Given 3rd med.cycle
Cases-
Given 3rd med.cycle
Cases-
Given 1st med. cycle

For the single-event survival analysis and the competing risks setting, the estimated restricted expected length of stay measure relative to experiencing the 1st antidepressant medication use can be interpreted as the expected medication-free time/ or time before experiencing the 1st antidepressant medication (Figure A3a). Regarding the 3-state Illness-Death approach, the interpretation can be either the expected time before experiencing the 1st antidepressant medication or the life expectancy after experiencing the 1st antidepressant medication. Under the 4-state unidirectional multi-state model, the estimated restricted expected length of stay can be interpreted as the length of stay in the 1st medication cycle before moving on to the 1st discontinuation period or death. Even though BC cases appear to stay in the 1st antidepressive medication cycle longer than the BC-free individuals, it can be observed that both groups do not tend to stay in the 1st medication cycle for a long period. As shown in Figure 8 of the main manuscript (sensitivity analysis), the measures derived from the 4-state unidirectional approach are heavily influenced from the definition of the medication cycle. Under the 4-state bidirectional multi-state (Figure A3b) the restricted expected length of stay in a medication cycle or medication discontinuation period after entering a medication discontinuation state is derived. As discussed above, this structure assumes same transition rates towards medication cycles (or medication discontinuation periods), irrespective of past transitions to those states. Under the recurrent multi-state structure without and with restrictions (“Emulated” bidirectional model), Figure A3c depicts the total restricted expected length of stays in a medication cycle given that an individual just entered her 1st, 2nd or 3rd medication cycle.

Figure A4. Comparison of the estimate of populational total probability of being in a medication cycle for different definitions of the medication cycles (3 months versus 4 months versus 5 months) under the different clock approaches for the multistate structures D, E, F and G.

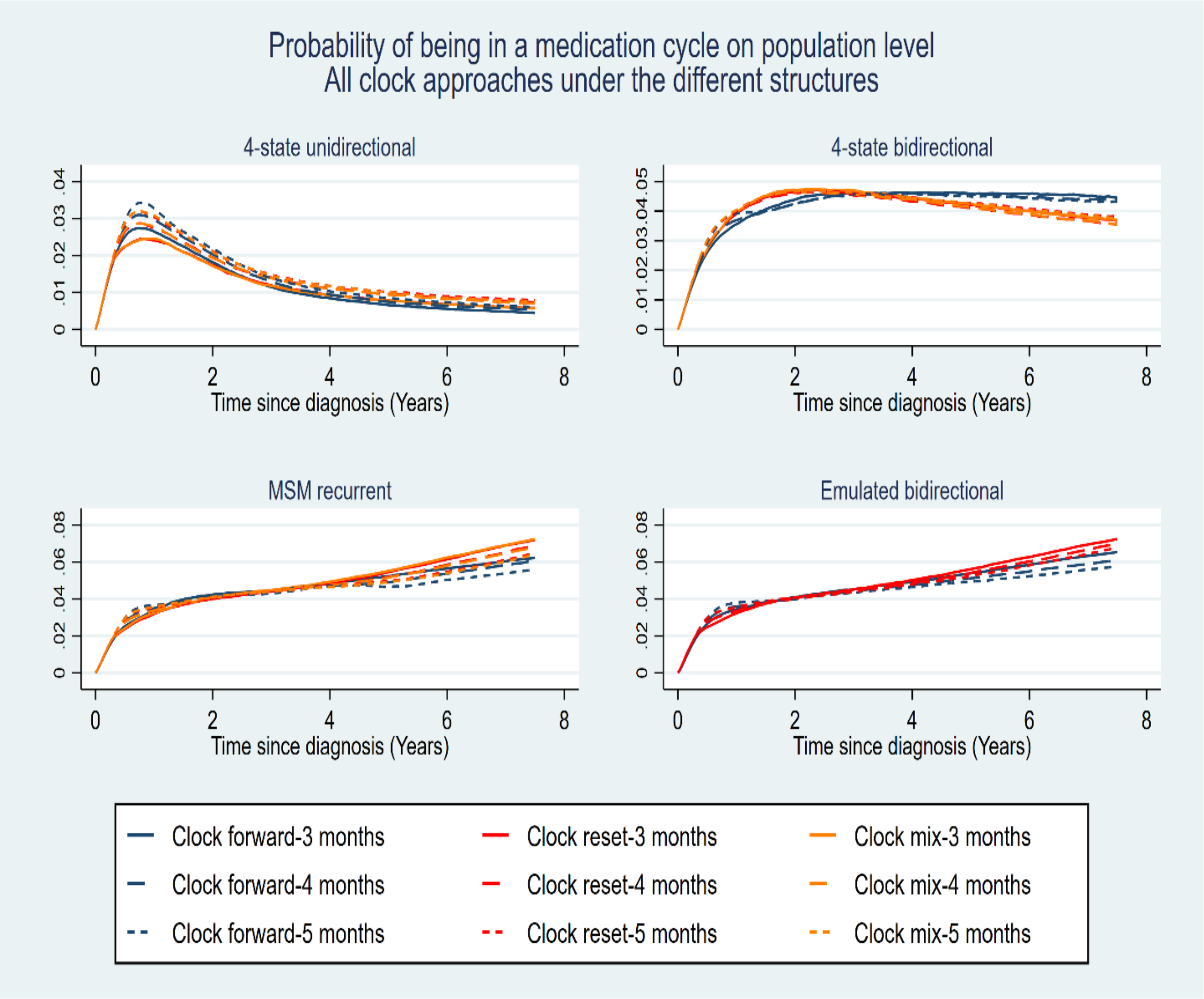

Several conclusions can be drawn from the estimated probabilities of FigureA4. Regarding the 4-state unidirectional model, the estimations from the three alternative clock approaches within each medication cycle definition seem to be very similar. However, as was also shown and discussed in Figure 8 of the main manuscript, the definition of the medication cycle influences the estimated probability of being in the 1st medication cycle, and this can be observed under all clock approaches. The 4-state bidirectional appears to be less sensitive to the definition of medication cycles. The Clock reset and Clock mix approaches return very similar probability estimates of being in a medication cycle under all medication cycle definitions. However, the Clock forward approach of this structure appears to be more sensitive to the medication cycle definition from times more than 4 years after the start of the follow-up and its probability estimates are not so similar with the ones derived from the Clock reset and Clock mix approaches. The estimated total probability of being in a medication cycle state since the start of follow-up is very similar among all Clock approaches and all medication cycle definitions. The "Emulated bidirectional" structure appears to have low to mild degree of sensitivity to the definition of the medication cycle and the choice of the clock approach used.

Flexible parametric survival models

In this study we used flexible parametric survival models on the log cumulative hazard scale $\ln(H)$ with time since entering a state as the timescale t. These models use restricted cubic spline functions g to flexibly model the effect of the logarithm of the timescale, $g(\ln t|\boldsymbol{\gamma}, \boldsymbol{m_0})$ for the log baseline cumulative hazard, with $\boldsymbol{m_0}$ knots and parameters $\boldsymbol{\gamma}$. The case status variable is included as X_i, the vector of the age group binary covariates as $\boldsymbol{Z_i}$ and their interactions as $X_i\boldsymbol{Z_i}$.

For the transitions towards the death state (medication cycle towards death, medication discontinuation period towards death) a model with 4 df for the baseline hazard is used and no time-dependent effects of the case status variable:

$$\ln[H(t|X_i, \boldsymbol{Z_i})] = g(\ln t|\boldsymbol{\gamma}, \boldsymbol{m_0}) + \beta_X X_i + \boldsymbol{\beta_Z Z_i} + \boldsymbol{\beta_{XZ}} X_i \boldsymbol{Z_i} \quad (1)$$

with β_X the coefficient for the covariate X, and $\boldsymbol{\beta_Z}$ the coefficient vector for the age group covariate vector $\boldsymbol{Z}$ and $\boldsymbol{\beta_{XZ}}$ the coefficient vector of their interaction.

For the transitions towards a non-death state (medication cycle towards medication discontinuation period and vice versa), a model with 4 df for the baseline hazard and $D = 3$ (number of time-dependent effects) for the case status variable is used:

$$\ln[H(t|X_i, \boldsymbol{Z_i})] = g(\ln t|\boldsymbol{\gamma}, \boldsymbol{m_0}) + \beta_X X_i + \boldsymbol{\beta_Z Z_i} + \boldsymbol{\beta_{XZ}} X_i \boldsymbol{Z_i} + \sum_{d=1}^{D=3} g\left(\ln t \middle| \boldsymbol{\delta_m}, \boldsymbol{m_j}\right) X_i \quad (2)$$

with, $\boldsymbol{m_j}$, the knots for the dth time-dependent effect with parameters $\boldsymbol{\delta_m}$.

Multi-state structure with recurrent couples of medication cycle and discontinuation period states (Corresponds to Structures of Figure 1F and 1G)

Let's consider a stochastic process $Y(t)$ with state space $\Omega = 1, \ldots, L$, with State 1 being the starting state of the process (start of follow-up) and L being the terminal state (Death). According to the recurrent multi-state structure of of Figures 1F and 1G, the even numbered states, $A = \{2, 4, \ldots, L-1\}$ are the medication cycle states (states of primary interest), with a_j the jth element of set A, and $j \in J_A = \{1, 2, \ldots, N_A\}$, N_A being the number of medication cycle states. The uneven states, $B = \{3, 5, \ldots, L-2\}$ is the of discontinuation period states, with b_k being the kth element of set B. Let t be the time of prediction (time since start of follow-up), and r_j the time of entering the jth medication cycle. We are interested only in estimates either since the start of the follow-up ($s = 0$) or immediately upon entering the jth medication cycle (estimates truncated at r_j).

We can define the total probability of being in any of the medication cycle states (set of states A) up to time t since since the start of follow-up as:

$$P(Y(t) \in A \,|\, Y(0) = 1) = \textstyle\sum_{j \in J_A} P\left(Y(t) = a_j \middle| Y(0) = 1\right) \quad (3)$$

The probability of being in the jth medication cycle up to time t given entering it at r_j, can be defined as:

$$P\big(Y(t) = a_j \big| Y(r_j) = a_j\big) \tag{4}$$

Let's now split the set of states of interest in two subsets based on the jth medication cycle, with $A_{j^-} = \{a_{j^-}, j^- \in J^-\}$ being the subset of all medication cycles before the jth cycle and $A_{j^+} = \{a_{j^+}, j^+ \in J^+\}$ being the subset of all medication cycles from the jth cycle and all subsequent cycles, with $J^- = \{1, \dots, j-1\}$ and $J^+ = \{j, \dots, N_A\}$.
We can then define the total probability of being in the jth medication cycle or any subsequent medication cycle across time t since start of follow-up given entering the jth cycle at time r_j as:

$$P\big(Y(t) \in A \big| Y(r_j) = a_j\big) = P\big(Y(t) \in A_{j^+} \big| Y(r_j) = a_j\big) = \sum_{j^+ \in J^+} P\big(Y(t) = a_{j^+} \big| Y(r_j) = a_j\big) \tag{5}$$

By integrating the probability defined in equation 3 from 0 to time t since the start of the follow-up, we can define the total restricted expected length of stay in all medication cycle states (set A) until time t:

$$\int_0^t \sum_{j \in J_A} P\big(Y(u) = a_j \big| Y(0) = 1\big)\, du \tag{6}$$

We can similarly define the total restricted expected length of stay in the jth medication cycle and all subsequent medication cycles across time t given entering the jth cycle at time r_j, by integrating from s to t the transition probability of equation 5:

$$\int_0^t \sum_{j^+ \in J^+} P\big(Y(u) = a_{j^+} \big| Y(r_j) = a_j\big)\, du \tag{7}$$

As in the main analysis of the study we use a semi-Markov ("clock reset") multi-state model, the predictions (transition probabilities and restricted expected length of stay measures) made on the time since start of follow-up t given entering the jth medication cycle state at time r_j, can also be reported as predictions made from time since entering each medication cycle state state up to $t - r_j$.
